# Draft Genome Sequences of *Listeria monocytogenes* Strains from Listeriosis Outbreaks Linked to Soft Cheese in Washington State

**DOI:** 10.1128/genomeA.00936-17

**Published:** 2017-09-07

**Authors:** Zhen Li, Paula A. Marsland, Roxanne T. Meek, Kaye Eckmann, Marc W. Allard, Ailyn C. Pérez-Osorio

**Affiliations:** a Washington State Department of Health, Public Health Laboratories, Shoreline, Washington, USA; b Division of Microbiology, Office of Regulatory Science, Center for Food Safety and Nutrition, U.S. Food and Drug Administration, College Park, Maryland, USA

## Abstract

*Listeria monocytogenes* has caused listeriosis outbreaks linked to soft cheese. Here, we report the draft genome sequences of seven *L. monocytogenes* isolates from two possibly related outbreaks caused by soft cheese products in Washington State.

## GENOME ANNOUNCEMENT

*Listeria monocytogenes* is a ubiquitous, Gram-positive, non-spore-forming, rod-shaped, motile aerobic, and facultatively anaerobic bacterium (1). Through contaminated food products, *L. monocytogenes* causes high-mortality foodborne illnesses in immunocompromised individuals, particularly children and pregnant women. Listeriosis caused by *L. monocytogenes* infection can lead to miscarriage, encephalitis, and septicemia (2). Recently, multiple *L. monocytogenes* outbreaks have been linked to Mexican-style soft cheese in the United States (3, 4). Between 2009 and 2010, an *L. monocytogenes* outbreak associated with a Mexican-style soft cheese product (brand A) was identified in Washington State. In late 2014, we noticed the recurrence of clinical *L. monocytogenes* isolates with the same pulsed-field gel electrophoresis (PFGE) pattern as strains collected in the 2009–2010 outbreak. During the follow-up investigation, a whole-genome sequencing approach was used to determine that four environmental and food isolates from the 2009–2010 outbreak and three clinical isolates from 2014 were linked.

Listeria DNA was extracted using a DNeasy blood and tissue kit (Qiagen, Valencia, CA) and prepared with an Illumina Nextera XT kit. The 250-bp paired-end sequencing was performed using an Illumina MiSeq sequencer. Raw sequencing reads were quality trimmed using Trimmomatic (version 0.36) (5). *De novo* assembly of trimmed reads was performed using SPAdes (v 3.10.0) (6). The NCBI Prokaryotic Genome Automatic Annotation Pipeline (PGAAP) was used to annotate the *Listeria* genomes (7). Sequence types (ST) were determined using a multilocus sequence typing (MLST) subtyping method at the Institut Pasteur MLST database (http://bigsdb.web.pasteur.fr/listeria/listeria.html). The PCR serogroups were also determined at the Institut Pasteur MLST database. Whole-genome MLST (wgMLST) analysis was done using BioNumerics (v 7.6) to compare similarity among the isolates. Single nucleotide polymorphism (SNP) analysis was performed using the FDA SNP Pipeline (8) and an NCBI reference *L. monocytogenes* genome (NC_003210).

The number of bases from raw fastq files ranged from 258.1 million to 635 million. These seven isolates had contig numbers from 13 to 15 (>500 bp) with a contig *N*_*50*_ value of 349,495 bp. The average coverage was 54× to 147× for all samples. The G+C content was 37.9%, and the genome size was 2.88 Mb from all the samples. Based on the annotation results, the numbers of total coding DNA sequences (CDS) were from 2,852 to 2,866, and the numbers of coding genes were from 2,826 to 2,840. MLST analysis indicated that all the isolates belonged to lineage I, sequence type 663, clonal complex 663, and PCR serogroup IVb. The wgMLST analysis showed a median allele difference of 7 (range, 0 to 9) among the isolates. SNP analysis showed 3 to 5 SNP differences among the three clinical samples from 2014, while 4 to 12 SNP differences were found among the food and environmental isolates from 2009. The SNP differences between the two groups were from 9 to 18 SNPs, indicating a potential association between the two outbreaks. Further analysis will be performed to scrutinize differences among these closely related isolates.

### Accession number(s).

This whole-genome shotgun project has been deposited at DDBJ/ENA/GenBank. Accession numbers and other relevant information are listed in Table 1. The versions of GenBank accession numbers described in this paper are the first versions.

**TABLE 1:** *L. monocytogenes* isolates described in this study

Isolate name	Yr isolated	Specimen type	BioSample name	SRA^a^ accession no.	GenBank accession no.
PNUSAL001227	2014	Blood, NOS^b^	SAMN03265978	SRR1734294	NKVU00000000
PNUSAL001228	2014	Blood, NOS	SAMN03265979	SRR1734295	NKVT00000000
PNUSAL001229	2014	Blood, NOS	SAMN03265980	SRR1734296	NKVS00000000
WAPHL_LIS_A00031	2010	Environmental swabs	SAMN03339952	SRR1805608	NKVY00000000
WAPHL_LIS_A00032	2010	Cheese	SAMN03339953	SRR1805602	NKVX00000000
WAPHL_LIS_A00033	2010	Environmental swabs	SAMN03339954	SRR1805571	NKVW00000000
WAPHL_LIS_A00034	2010	Environmental swabs	SAMN03339955	SRR1805592	NKVV00000000

a SRA, Sequence Read Archive.

b NOS, not otherwise specified.
